# Integrated machine learning approaches for flow cytometric quantification of myeloid-derived suppressor cells in acute sepsis

**DOI:** 10.3389/fimmu.2022.1007016

**Published:** 2022-11-17

**Authors:** Anthony S. Bonavia, Abigail Samuelsen, Joshua Luthy, E. Scott Halstead

**Affiliations:** ^1^ Division of Critical Care Medicine, Department of Anesthesiology and Perioperative Medicine, Penn State Milton S. Hershey Medical Center, Hershey, PA, United States; ^2^ Department of Anesthesiology and Perioperative Medicine, Penn State Milton S. Hershey Medical Center, Hershey, PA, United States; ^3^ Product Innovation Division, BD Life Sciences - FlowJo, Ashland, OR, United States; ^4^ Division of Pediatric Critical Care Medicine, Department of Pediatrics, Penn State Milton S. Hershey Medical Center, Hershey, PA, United States

**Keywords:** high parameter flow cytometry, sepsis - diagnostics, clustering analysis, clinical immunology, myeloid-derived suppressor cells (MDSC)

## Abstract

Highly heterogeneous cell populations require multiple flow cytometric markers for appropriate phenotypic characterization. This exponentially increases the complexity of 2D scatter plot analyses and exacerbates human errors due to variations in manual gating of flow data. We describe a semi-automated workflow, based entirely on the Flowjo Graphical User Interface (GUI), that involves the stepwise integration of several, newly available machine learning tools for the analysis of myeloid-derived suppressor cells (MDSCs) in septic and non-septic critical illness. Supervised clustering of flow cytometric data showed correlation with, but significantly different numbers of, MDSCs as compared with the cell numbers obtained by manual gating. Neither quantification method predicted 30-day clinical outcomes in a cohort of 16 critically ill and septic patients and 5 critically ill and non-septic patients. Machine learning identified a significant decrease in the proportion of PMN-MDSC in critically ill and septic patients as compared with healthy controls. There was no difference between the proportion of these MDSCs in septic and non-septic critical illness.

## Introduction

Myeloid-derived suppressor cells (MDSCs) are a heterogeneous population of myeloid cells that suppress T cell and natural killer cell activity. Previously known as “natural suppressor” cells, these cells are believed to be central to the pathogenesis of cancer, where they cause immune dysfunction and resilience to chemotherapeutic agents (1, 2). More recently, MDSCs have also been implicated in the pathophysiology of sepsis, the life-threatening organ dysfunction that results from a dysregulated host immune response to infection (3, 4). However, MDSCs may also play a protective role during hyperinflammatory disease processes (5).

The rapid progression of sepsis leaves a narrow but critical time window in which clinicians can potentially intervene to improve patient outcomes. MDSCs have been proposed as a therapeutic target in this window (3), although investigations into their pathophysiology have met several challenges. First, the current gold standard for quantifying MDSC number and/or function involves a T cell proliferation assay which measures their suppressive activity over several days. This delay impacts our ability to intervene early in sepsis *via* the administration of immune adjuvants to patients who may benefit from this therapy. Second, sepsis is a highly heterogeneous syndrome marked predominantly by hyper-inflammation in certain patients and immune paralysis in others, but often by both processes in concert (6, 7). It is likely that both MDSC number and function are equally heterogeneous in these different sepsis subtypes. Murine MDSCs are ubiquitously identified as CD11b^+^ Ly6G^+^ Ly6C^lo^ (PMN-MDSC) or CD11b^+^ Ly6G^-^ Ly6C^hi^ (monocytic, or M-MDSC). In contrast, there is tremendous inconsistency in the nomenclature and surface markers that characterize human MDSCs. Despite general guidelines designed to minimize bias in the presentation and interpretation of flow cytometric data, significant variations between individuals and laboratories persist and may affect the conclusions drawn from flow cytometry data (8, 9).

We hypothesized that the integration of supervised and unsupervised clustering analyses, using machine learning algorithms, would highlight MDSC subpopulations that correlate with patient outcomes. We investigated our hypothesis by (1) comparing MDSC quantification by using this approach with results obtained using manual gating alone, and (2) examining the relationship between the number of MDSCs calculated by each method and patient outcomes. We completed all analyses by using the Flowjo Graphical User Interface (GUI) to demonstrate that meaningful machine learning approaches involving large flow cytometric data sets does not require extensive knowledge of bioinformatics or computer programming.

## Methods

### Study participants

This prospective, observational trial was performed on critically ill, adult patients and healthy control volunteers, between 11/2021 and 6/2022. Hospitalized patients were screened for potential sepsis, based on Modified Early Warning Score-Sepsis (MEWS-Sepsis) Recognition Score (10–12), by using a computational algorithm executed twice daily. Two investigators then independently assessed each identified patient for study inclusion according to Sepsis-3 criteria (4). Specifically, sepsis was defined as a change in sequential organ failure assessment (SOFA) score of two or more in the setting of clinically suspected or microbiologically-proven infection. Critical illness was defined as the need for continuous intravenous infusion of vasopressors to maintain a mean arterial pressure of ≥65 mmHg, and/or the need for continuous respiratory support and monitoring, and/or the need for continuous renal replacement therapy. Critically ill and non-septic patients included adult patients who were older than 18 years and fulfilled criteria for critical illness but not sepsis. Healthy volunteers included non-hospitalized adults that did not have major medical comorbidities, did not take immune-suppressive medications, and did not have known immunologic disorders. To minimize the potential or confounding effects, we excluded patients with active hematologic malignancies, autoimmune disorders and those who were receiving immunomodulating therapies. All participants provided informed consent in accordance with the institutional Human Study Protection Office (Protocols #15328 and #10357).

### Clinical variables

Patient data for critically ill, septic (CIS) and non-septic (CINS) patients was obtained from the electronic medical record. We utilized the Charlson Comorbidity Index, Acute Physiology and Chronic Health Evaluation (APACHE II) and Sequential Organ Failure Assessment (SOFA) scores to classify illness severity (13–16).

### Processing of blood samples

Venous blood samples were collected in tubes containing ethylenediamine tetra-acetic acid (EDTA), within 24h of the onset of critical illness +/- sepsis (day 1). Complete blood count was assessed by the institution’s clinical laboratory. Furthermore, 100µl of whole blood was blocked with mouse serum (0.5:1, M5905, Sigma-Aldrich, St. Louis, MO), for 5 minutes at room temperature followed by addition of e780 Fixable Viability Dye (1:800, cat #65-0865, eBioscience, San Diego, CA). The following antibodies were then added for 20 minutes at room temperature, in the dark: anti-CD66b-FITC (1:20, cat #555724, BD Biosciences, San Diego, CA), anti-CD115-PerCP-Cy5.5 (1:200, clone 9-4D2-1E4, #347309, Biolegend, San Diego, CA), anti-CD16-BV421 (1:80, clone 368, #562874, BD), anti-CD11b-BV605 (1:80, clone ICRF44, #301332, Biolegend), anti-HLA-DR-BV650 (1:80, clone L243, #307649, Biolegend), anti-CD15-BV711 (1:80, clone W6D3, #563142, BD), anti-CD14-BV786 (1:80, clone M5E2, #563699, BD), anti-Lineage Cocktail CD3/19/20/56-APC (1:20, #363601, Biolegend), anti-CD45-AF700 (1:80, clone 2D1, #368513, Biolegend), anti-CD33-PE (1:20, #555450, BD), anti-CD123-PE-Cy7 (1:80, clone 7G3, #560826, BD). This antibody panel was created following a literature search into recently published strategies for identifying human MDSCs (17–21).

Following antibody staining, red blood cells were lysed, and leukocytes were washed and fixed. Counting Beads (Invitrogen, Waltham, MA) were added to a separate sample of lysed, unstained cells. Analysis was performed on FACSymphony A3 (Becton Dickinson & Company, Franklin Lakes, NJ) and using Flowjo v10.8.1 (BD Biosciences). Instructions for installing and running each plugin *via* the Flowjo GUI is found in the Supplementary Material.

### Manual gating of flow cytometric data

**Figure 1:**
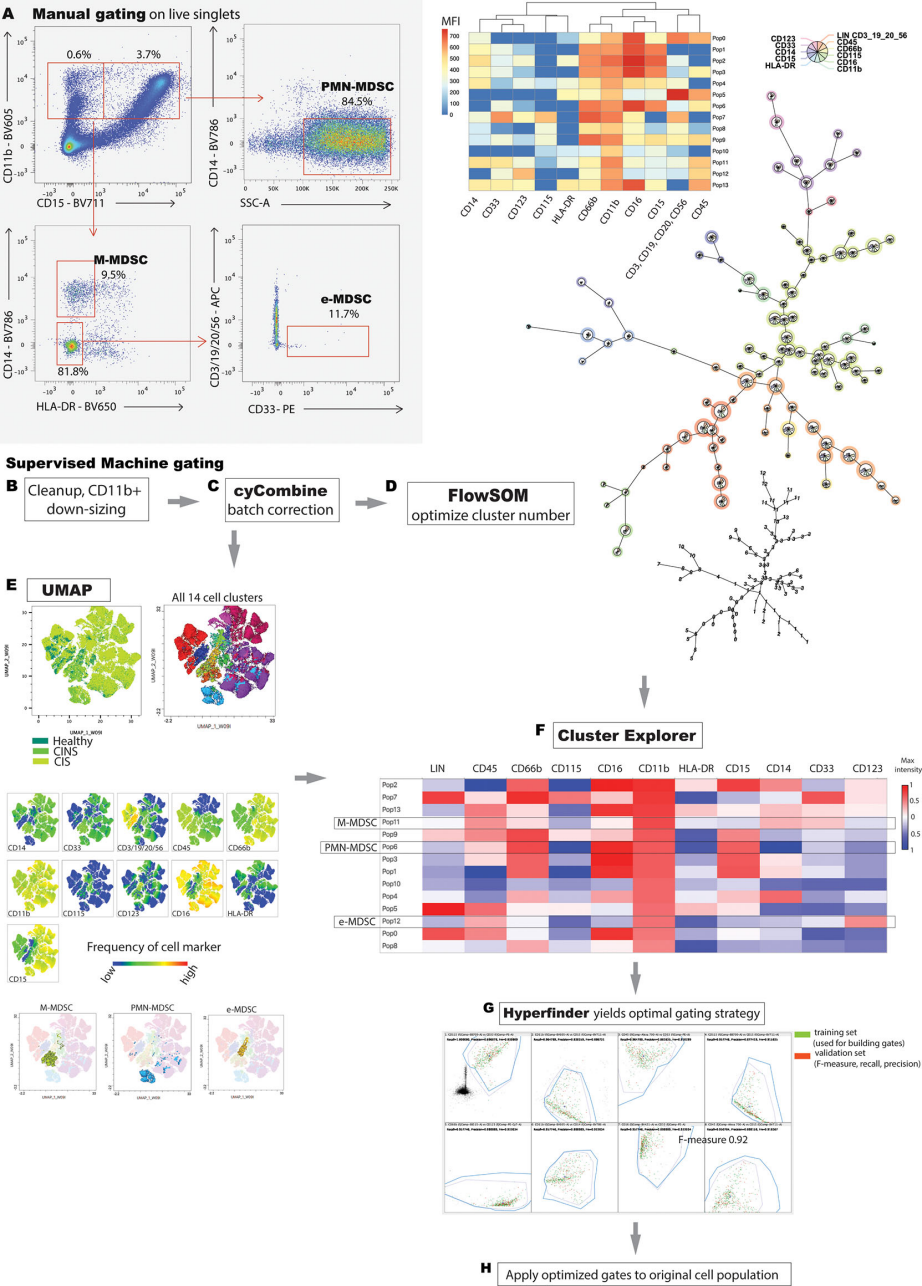
Manual versus machine gating of MDSC populations. **(A)** Representative manual gating strategy used to identify MDSC. M-MDSCs are CD11b^+^, HLA-DR^-^, CD15^-^, CD14^+^; PMN-MDSCs are CD15^+^, CD14^-^, CD11b^+^, SSC^hi^; e-MDSCs are CD14^-^, CD15^-^, CD3^-^, CD19^-^, CD20^-^, CD56^-^, HLA-DR^-^, CD33^+^, CD11b^+^. **(B)** Stepwise integration of supervised, machine learning techniques facilitates analysis of high parameter flow cytometric data. Rudimentary gating isolates viable singlets, and subsequent manual gating for the ‘lowest common denominator’ (CD11b+ surface marker, in this case) enriches the population of interest. Downsizing to a minimal, common cell count eliminates sampling bias. **(C)** cyCombine corrects for technical artifacts arising from different experiments. **(D)** User optimizes number of FlowSOM clusters to appropriately fit the model to the data set. FlowSOM, generates a minimal spanning tree and illustrates marker positivity of each identified cluster. **(E)** Dimensionality reduction allows two-dimensional data representation while preserving the global data structure. Proximal clusters on the 2-dimensional plot have closely related surface marker profiles. **(F)** FlowSOM and UMAP data are integrated in Cluster Explorer, allowing in-depth data exploration and cluster interrogation. PMN-MDSC, M-MDSC and e-MDSC were determined based on the definitions established in A, and by using a conservative estimate of >10% of maximum positive/negative intensity to denote marker positive/negative status respectively. Unannotated cell populations do not meet criteria for M-, PMN- or e-MDSC. **(G)** Hyperfinder optimizes polygon gating of populations of interest. **(H)** The Hyperfinder algorithm is then applied to flow data from step **(B)** Displayed is an example of Hyperfinder gating for e-MDSC cluster.

MDSCs nomenclature was consistent with recently described minimal phenotypic characteristics necessary to identify cells as MDSC (22) and with nomenclature utilized in a comparable study of septic patients (21). M-MDSCs were CD11b^+^, CD15^–^, CD14^+^, HLA-DR^–^; PMN-MDSCs were CD15^+^, CD14^–^, CD11b^+^, SSC^hi^; early (e)-MDSCs were CD3^–^, CD14^–^, CD15^–^, CD19^–^, CD56^–^, HLA-DR^–^, CD33^+^, CD11b^+^. Flow data was analyzed, and manual gating performed and presented, in accordance with guidelines for the use of flow cytometry in immunologic studies (8) (**Figure 1A**).

### Machine clustering of flow cytometric data

A comprehensive literature search revealed that the only between-study consistency in human MDSC characterization is the pan-myeloid, CD11b^+^ surface marker. Thus, we pre-processed flow data by first applying bead compensation and then by manually gating for viable, CD11b^+^ singlets using Flowjo 10.8.1 (BD Biosciences) (**Figure 1B**). Flowjo is a GUI, used to launch the plugins detailed below. We employed a ‘lowest common denominator’ approach to minimize bias, to preserve integrity of the dataset and to rely predominantly on machine learning for subsequent exploration. 2D scatter plots clearly delineated between CD11b^-^ and CD11b^+^ populations in all samples (**Supplementary Figure 1**). We then used the Flowjo Downsample plugin v3.3.1 to export an equal number of normally distributed CD11b^+^ events from each sample for subsequent processing (7000 events in this case).

Given the stepwise collection of blood samples over the study period, batch correction was performed by using cyCombine, a plugin running in the R programming language (**Figure 1C**) (23). CyCombine integrates cytometry data from different experiments, maintaining the biologic variance and structure of data while minimizing dataset differences due to technical variance (23). Supervised clustering was then performed by FlowSOM v3.0.18 (R language plugin), a self-organizing map algorithm that provides visual representation of data by using a minimal spanning tree (24) (**Figure 1D**). Different cluster numbers were selected and the output was analyzed by the operator to determine appropriate fitting to the dataset.

Uniform Manifold Approximation and Projection (UMAP v3.1, Python) was used for dimensionality reduction (25) (**Figure 1E**). UMAP is a scalable machine learning algorithm that creates an intuitive, 2-dimensional map wherein spatial proximity of clusters implies similar cell marker phenotype.

### Comparison of clusters, automated gating and iterative application to samples

ClusterExplorer v1.6.6 allows the integration of one or more clustering strategies with user-defined surface markers of interest and dimensionality reduction x and y parameters. We selected ClusterExplorer heat map as the primary tool with which identify discrete MDSC populations, based on the intensity of different surface markers as they relate to *a priori* definitions of each of the three MDSC sub-types (**Figure 1F**). ClusterExplorer can present data on profile graphs with relative expression levels of surface markers, and in bar charts showing the relative numbers of events in each cluster.

After identifying MDSC subsets, Hyperfinder v0.6.8 (Java) was used to optimize the gating strategy for each identified cell population (maximum of 8 gates, target F-measure beta of 1) (**Figure 1G**). F-measure is the harmonic mean of yield and purity, with a score of 1 indicating equal contributions of yield and purity. The resulting gating algorithm for each MDSC subset was then retroactively applied to the original CD11b^+^ population from each sample (**Figure 1H**). The number of cells identified by this approach that met criteria for e-MDSC, M-MDSC and PMN-MDSC was compared with equivalent populations of cells identified by manual gating.

### Statistical analyses

Analysis was performed in Prism v.9.3.1 (Graphpad Software, San Diego, CA), with statistical details contained within the respective figure legends.

## Results

We screened 1750 patients identified by MEWS-Sepsis criteria at a tertiary care academic medical center, of which 1596 did not meet inclusion criteria for sepsis and critical illness, and 128 patients met study exclusion criteria. Our study cohorts consisted of 17 critically ill and septic (CIS) patients, 5 critically ill and non-septic (CINS) patients and 5 healthy volunteers. This convenience sample was selected based on the importance of MDSCs in the pathophysiology of CIS patients, combined with the severity of illness and high mortality rate experienced by these patients. The data of one septic patient was excluded from analysis due to profound leukopenia and insufficient number of cells for flow cytometric analysis. **Table 1** compares the demographic profile of CIS and CINS patients. The mean age of healthy volunteers was 43 years (range 24-63) and 80% were male. One was Asian, another Hispanic and three Caucasian.

**Table 1:** Patient demographics and outcomes.

	Critically ill, septic (n=17)	Critically ill, non-septic (n=5)
Age, mean (range)	71 (31–91)	76 (59–88)
Female	12 (71%)	2 (40%)
Septic shock on admission	3 (18%)	NA
***Comorbidities***		
Cancer	6 (35%)	1 (20%)
Cardiovascular disease	8 (47%)	4 (80%)
Peripheral vascular disease	2 (12%)	1 (20%)
Diabetes	7 (41%)	1 (20%)
Gastrointestinal disease	6 (35%)	3 (60%)
Hepatic disease	0	1 (20%)
Hypertension	9 (53%)	4 (80%)
Kidney or Urologic disease	4 (24%)	3 (60%)
Cerebrovascular or Neurologic disease	5 (29%)	2 (40%)
Obesity	5 (29%)	1 (20%)
Respiratory Disease	4 (24%)	1 (20%)
Thyroid Disease	6 (35%)	1 (20%)
***Severity of Illness***		
APACHE II score	24 ± 7	28 ± 5
SOFA score	7 ± 3	8 ± 4
Charlson Comorbidity Index	6 ± 3	7 ± 2
***Laboratory Values***		
Leukocyte Count (x10^3^/µl)	21 ± 11	16 ± 11
Absolute lymphocyte count (x10^3^/µl)	0.8 ± 0.4	1.3 ± 1.2
Absolute monocyte count (x10^3^/µl)	0.7 ± 0.8	1.5 ± 0.9
Lactic acid (mg/dL) on admission	3 ± 2	3 ± 2
***Outcomes***		
Secondary infection rate	4 (23%)	0
In-hospital mortality rate	7 (41%)	1 (20%)
30-day mortality rate	7 (41%)	1 (20%)
Hospital length of stay (days)	11.9 ± 6.4	14.6 ± 4.7
Death-free days until follow-up (days)	60.2 ± 72	43.8 ± 36.7
ECOG/Zubrod Score at hospital discharge	3.4 ± 1.9	3.8 ± 0.8
ECOG/Zubrod Score at 30d	2.7 ± 1.2	3.0 ± 1.4
30-day hospital readmission rate	2 (12%)	0
Culture-positive sepsis rate	15 (88%)	NA
Total days of antibiotics within first month (days)	10.6 ± 7.4	4.4 ± 5.2
***Hospital Discharge Disposition***		
Discharged to home	7 (41%)	0
Discharged to nursing facility or to long-term acute care hospital	3 (15%)	5 (100%)

NA, Not Applicable.

**Figure 2:**
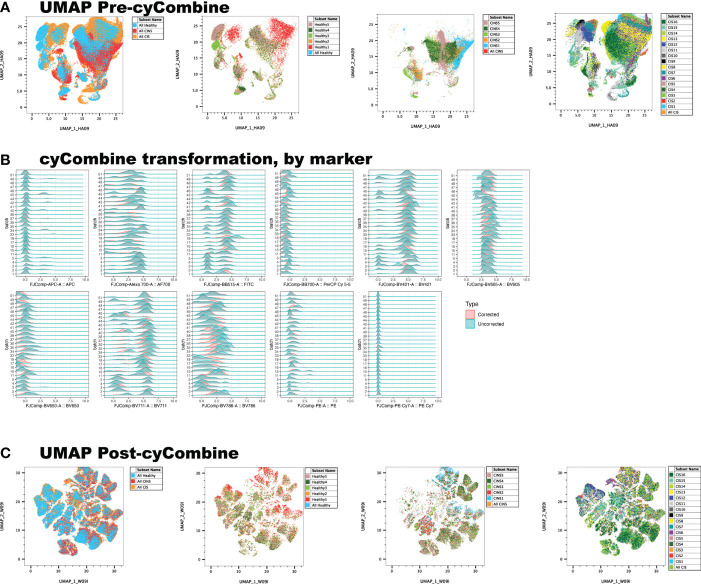
Correction of batch effects is an integral component of machine learning, as it minimizes data artifact due to technical variance between experiments. **(A)** UMAP representation of data reveals a homogenous, ‘streaky’ color pattern, characteristic of batch artifact. **(B)** Normalization of data allows the scalable, unbiased integration of multiple cytometry datasets for primary analysis. **(C)** cyCombine dramatically improves batch effect artifact.

**Figure 3:**
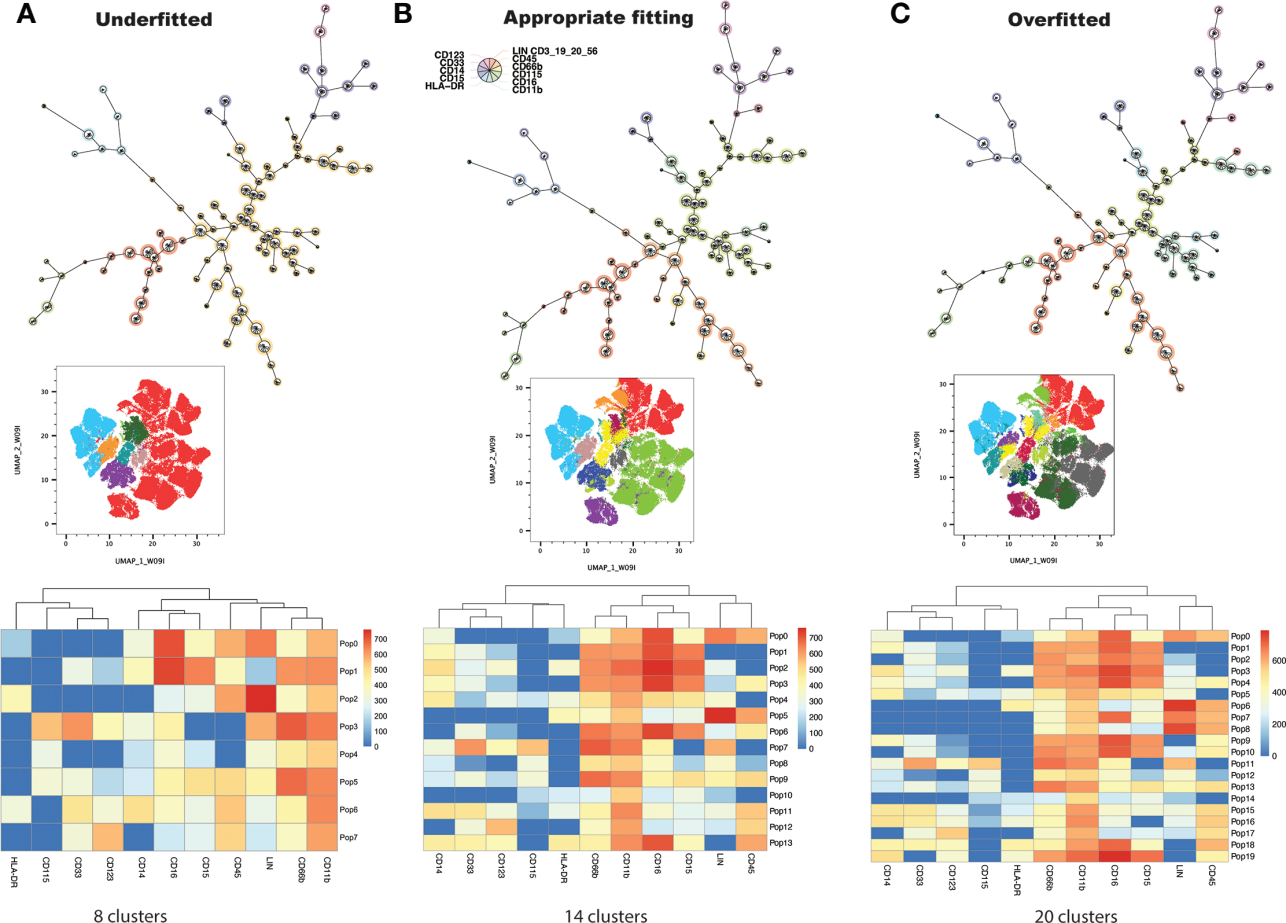
FlowSOM is repeatedly performed on the dataset, increasing cluster number at each iteration, and projecting clusters onto the dimensionally reduced, UMAP image at each iteration. The user determines the balance between underfitting **(A)** and overfitting **(C)** the model to the data, to derive a biologically meaningful cluster number **(B)**.

Conventional, manual gating of flow cytometric data was first performed (**Figure 1A**). Supervised machine learning and machine-optimized gating for PMN-MDSC, M-MDSC and eMDSC was run in parallel by using the strategy described in **Figures 1B–H**. CyCombine corrected for batch effects resulting from experiments spanning over an 8-month period (**Figure 2**). FlowSOM was repeated with cluster numbers varying from 8 to 24, and it identified 14 unique cell clusters as the optimum balance between underfitting and overfitting the model to the data (**Figure 3**). The UMAP transformation and 14-cluster FlowSOM model were then integrated by using ClusterExplorer, where user-driven exploration of the data revealed one likely M-MDSC metacluster, one e-MDSC cluster and one PMN-MDSC cluster that were consistent with the definitions established above.

Each of these three clusters was further interrogated by using the Hyperfinder tool, to determine the optimal flow gating strategy (**Figure 1G**). Hyperfinder reported gating F-measures of 0.97 for the e-MDSC cluster, 0.90 for the M-MDSC cluster and 0.98 for the PMN-MDSC cluster. **Supplementary Figure 2** juxtaposes the results of manual and machine gating strategies.

**Figure 4:**
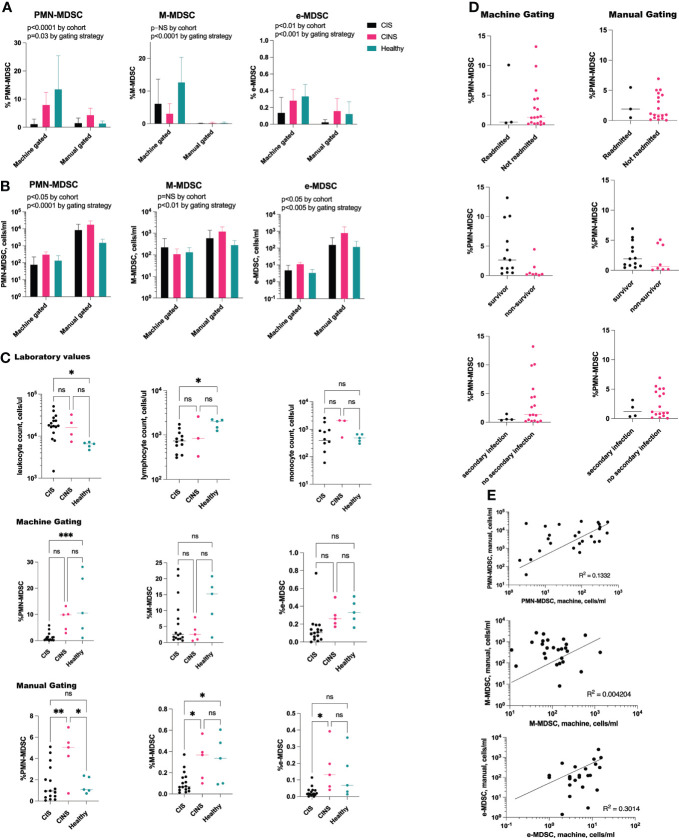
PMN-MDSCs and e-MDSCs are decreased in acute sepsis. **(A)** Two-way ANOVA demonstrates significant differences between the percentage of MDSCs by patient cohort. When controlling for gating strategy, PMN-MDSCs and e-MDSCs were significantly different between CIS, CINS and healthy patients (p=0.0006 and p=0.0013), while M-MDSCs did not differ. **(B)** These findings were consistent with absolute cell counts, where PMN-MDSCs and e-MDSCs differed (p=0.02 for both) although M-MDSCs did not differ between cohorts. **(C)** Despite higher leukocyte counts in CIS patients as compared with healthy patients, percentage of PMN-MDSCs was significantly decreased in sepsis, as assessed by machine gating. **(D)** Proportion of PMN-MDSCs did not predict 30-day clinical outcomes in CIS and CINS patients. **(E)** Positive correlation between MDSC population by manual gating versus machine learning. CIS, critically ill and septic (N = 16), CINS, critically ill and non-septic (N = 5). N for healthy donors = 5. ns, P > 0.05; *P ≤ 0.05; **P ≤ 0.01; ***P ≤ 0.001.

The number of MDSCs, as assessed by machine gating, correlated with but was significantly different from, that identified by manual gating alone (**Figures 4A, B**). When gating strategy was factored, using 2-way ANOVA, there remained a significant difference both in the number and in the percentage of PMN-MDSC and e-MDSC between CIS, CINS and healthy cohorts (**Figures 4B, C**). Interestingly, while total leukocyte count was significantly higher in CIS patients as compared with healthy volunteers (p=0.04), PMN-MDSCs by machine gating were significantly lower in CIS patients (p=0.0005), and PMN-MDSCs did not significantly differ between cohorts when assessed by manual gating alone. There was no difference between number of PMN-MDSCs in CIS and CINS cohorts, when assessed by a machine gating strategy (**Figure 4C**).

Neither manual nor machine gating demonstrated a correlation between MDSC number/percentage and 30-day clinical outcomes (hospital readmission rate, secondary infection rate, mortality rate) in CIS or CINS cohorts (**Figure 4D**). Linear regression analysis did not demonstrate a significant relationship between death-free days or hospital length of stay and PMN-MDSCs, M-MDSCs or e-MDSCs. There was a positive correlation between the number of MDSCs assessed by machine versus manual gating, although the R^2^ value was low (**Figure 4E**). Severity of illness scores (SOFA, APACHE II) did not appear to be related to number of MDSCs, whether assessed by manual or by machine gating.

## Discussion

Advanced flow cytometric tools have led to exponential increases in the size of datasets, the time taken to explore these datasets and the complexity of data analysis. This has created the imperative for efficient analytic tools that still allow meaningful human input. A recent study analyzed MDSCs derived from the peripheral blood mononuclear cells of septic patients, by using FlowSOM mapping (21). We present an alternative, semi-automated, supervised approach to identify MDSCs in whole blood. Our approach utilizes novel, complementary flow cytometric tools and could potentially be applied to future, prospective clinical trials that target highly heterogeneous cell populations.

The main strength of our study is its presentation of an exclusively GUI-based approach to multiparameter flow cytometric analysis, obviating the need for computer programming expertise. We are not advocating for complete automation of data analysis or for the removal of human input. Rather, we present a pipeline wherein complementary tools allow the exploration of large datasets accumulated over several experiments, while allowing user input at key junctures of the analysis. Our workflow also allows the simultaneous visualization of several cell markers, removing the need for multiple, pair-wise comparisons of individually standardized scatter plots.

While the size of our critically ill cohorts was small, our analysis did not corroborate our hypothesis that certain MDSC subpopulations correlate with 30-day clinical outcomes. However, our data revealed an interesting depression in the proportion of PMN-MDSC in the acute sepsis cohort, as compared with healthy patients. This decrease was observed at a time when leukocyte and neutrophil counts are typically elevated. This observation is especially significant since (1) we analyzed whole blood (rather than PBMC), and (2) it is therefore likely that a portion of these scant PMN-MDSCs are mature neutrophils, since CD11b^+^CD15^+^CD14^−^CD33^+/low^CD66b^+^ markers enrich for neutrophils at all maturation stages (10). High PMN-MDSCs within the first week of sepsis onset have been recently associated with secondary infections, while M-MDSCs appear to be more abundant in sepsis survivors (26). While MDSCs are known to expand and acquire their suppressive potential during protracted sepsis (13), there is limited human data about the kinetics and immune function of this population in early sepsis. If corroborated in a larger patient population, our findings may suggest that machine learning can detect subtle changes in MDSC subtypes before they are detectable by conventional flow gating approaches.

Our small sample size is reflective of the severity of illness required for our patients to meet study inclusion criteria (i.e., both sepsis and critical illness). While the population studied constitutes only a small proportion of all hospitalized patients, it accounts for a high healthcare burden, morbidity, and mortality. It is also the patient population in which expansion of MDSCs has been most extensively described, following acute infection (3, 17, 18, 27, 28). It is therefore imperative to study these patients and better understand their underlying pathophysiology.

Another limitation of our study is that we cannot, with certainty, conclude that the analyzed cell populations constitute ‘true’ MDSCs without ascertaining their cell-suppressive potential *in vitro*. Conversely one cannot quantify suppressive potential of a selected cell population without first isolating cells by their surface markers. The importance of assessing suppressive MDSC cell activity early in sepsis may be further obviated by recent evidence demonstrating that only MDSCs that are obtained at >14 days post-sepsis significantly suppress T lymphocyte proliferation and IL-2 production (13). Unlike murine studies, human MDSCs produced early in sepsis may have not yet adopted immunosuppressive properties, increasing the importance of accurate flow cytometric quantification of these cell populations.

Batch effects need to be accounted for when combining results obtained on different experimental days. Our analyses demonstrate an appropriate compensation for batch effects by using the cyCombine tool (23). CytoNorm is another Flowjo tool that is available for correction of batch effects. However, it requires the careful selection of experimental controls, which are challenging to obtain in a clinical study that recruits patients on an ongoing basis.

While not mandatory for the approaches described above, knowledge of the R programming language may allow further in-depth exploration of flow data sets. Github is a collaborative platform that hosts additional, R-based tools such as Milo, which is not yet available in the Flowjo GUI. Milo is a differential abundance testing tool that can reveal subtle cell state perturbations that are otherwise obscured by clustering approaches (29).

In conclusion, the semi-automated pipeline that we describe may be particularly useful when employing multiple flow parameters in the investigation of highly heterogeneous cell populations. It excels in the quantification of cell populations with *intermediate* phenotypes, and it is designed for use by immunologists having familiarity with Flowjo but minimal expertise in computer programming. The supervised approach allows users to provide input at key stages of the pipeline (e.g., selection of optimal cluster number), ensuring that the analysis remains biologically relevant and consistent with the goals of the investigation.

## Data availability statement

The raw data supporting the conclusions of this article will be made available by the authors, without undue reservation.

## Ethics statement

The studies involving human participants were reviewed and approved by Penn State Milton S. Hershey Medical Center, Institutional Review Board (IRB). The patients/participants provided informed consent to participate in this study.

## Author contributions

AB: Conceptualization, Methodology, Formal Analysis, Funding Acquisition, Investigation, Writing Original Draft, Review and Editing, Project Administration. AS: Investigation, Data Curation, Writing - Review and Editing. JL: Flowjo Technical Assistance. EH: Formal Analysis, Writing Original Draft, Reviewing and Editing. All authors contributed to the article and approved the submitted version.

## Funding

Funding was provided by the National Institute of General Medical Sciences #K08GM138825 (AB).

## Conflict of interest

JL is a paid employee of Becton, Dickinson and Company (BD Life Sciences), who has assisted us in the adaptation of cyCombine to the Flowjo GUI. He did not participate in funding, data acquisition, data analysis or writing of the manuscript.

The remaining authors declare that the research was conducted in the absence of any commercial or financial relationships that could be construed as a potential conflict of interest.

## Publisher’s note

All claims expressed in this article are solely those of the authors and do not necessarily represent those of their affiliated organizations, or those of the publisher, the editors and the reviewers. Any product that may be evaluated in this article, or claim that may be made by its manufacturer, is not guaranteed or endorsed by the publisher.

## Supplementary material

Supplementary Figure 1 Representative flow cytometric data illustrating CD11b-BV605 (y-axis) against cell viability (x-axis) in critically ill and septic, critically ill and nonseptic and healthy patients respectively. CD11b-BV605 fluorescence intensity >10^3^ was used as a cutoff value for CD11b expression, based on histogram data demonstrating a bimodal distribution separated at this cutoff value.

Supplementary Figure 2 Juxtaposition of MDSC clusters generated manually versus *via* supervised machine learning with FlowSOM algorithm followed by Hyperfinder gating.
