# Supplementary figures and images for: Integrated machine learning approaches for flow cytometric quantification of myeloid-derived suppressor cells in acute sepsis
Source: Front Immunol. 2022 Nov 17;13:1007016. doi: 10.3389/fimmu.2022.1007016 (PMC9714638; doi:10.3389/fimmu.2022.1007016)

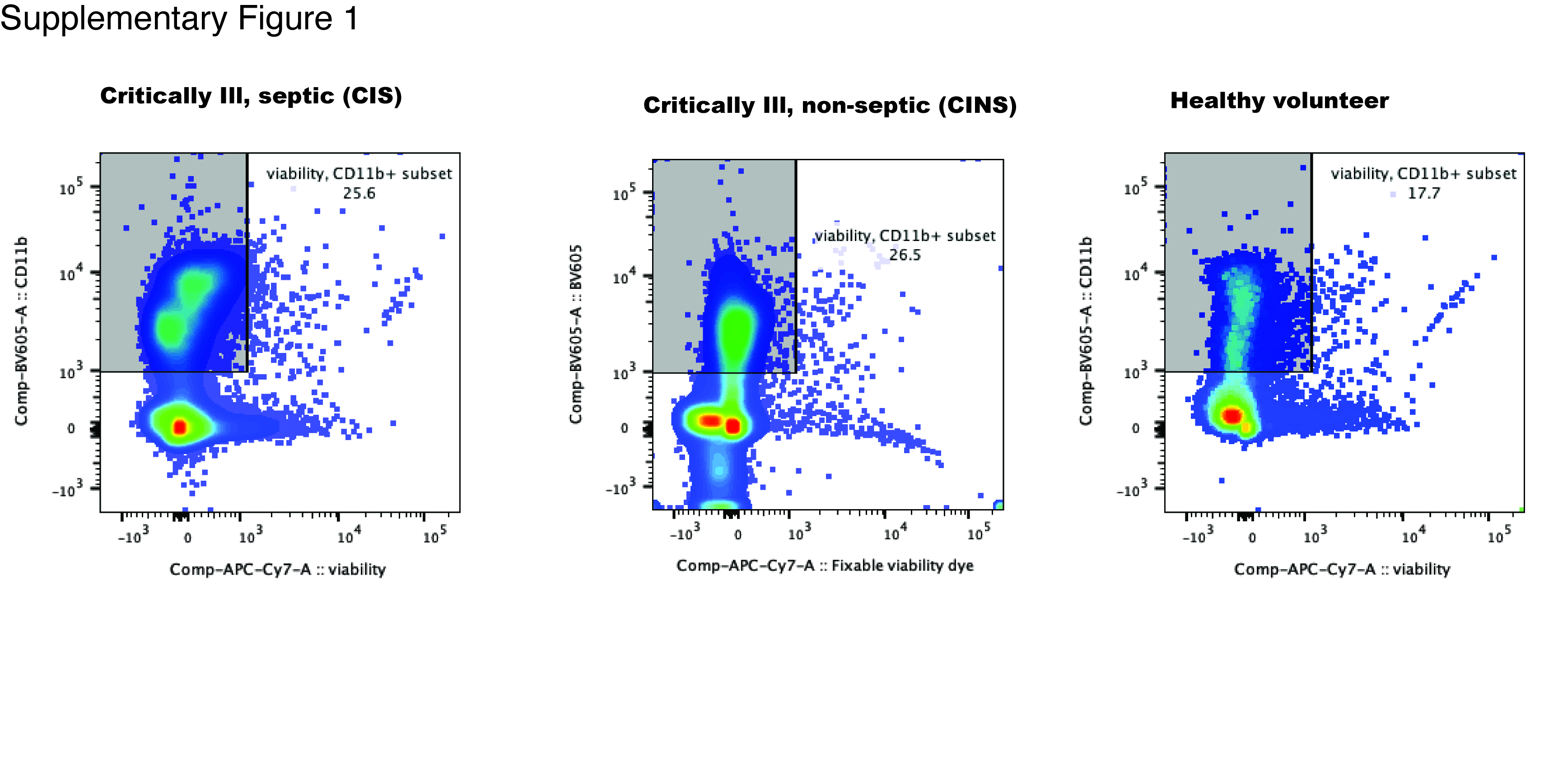

Supplement: Supplementary Figure 1 — Representative flow cytometric data illustrating CD11b-BV605 (y-axis) against cell viability (x-axis) in critically ill and septic, critically ill and nonseptic and healthy patients respectively. CD11b-BV605 fluorescence intensity >103 was used as a cutoff value for CD11b expression, based on histogram data demonstrating a bimodal distribution separated at this cutoff value. [file Image_1.tif]

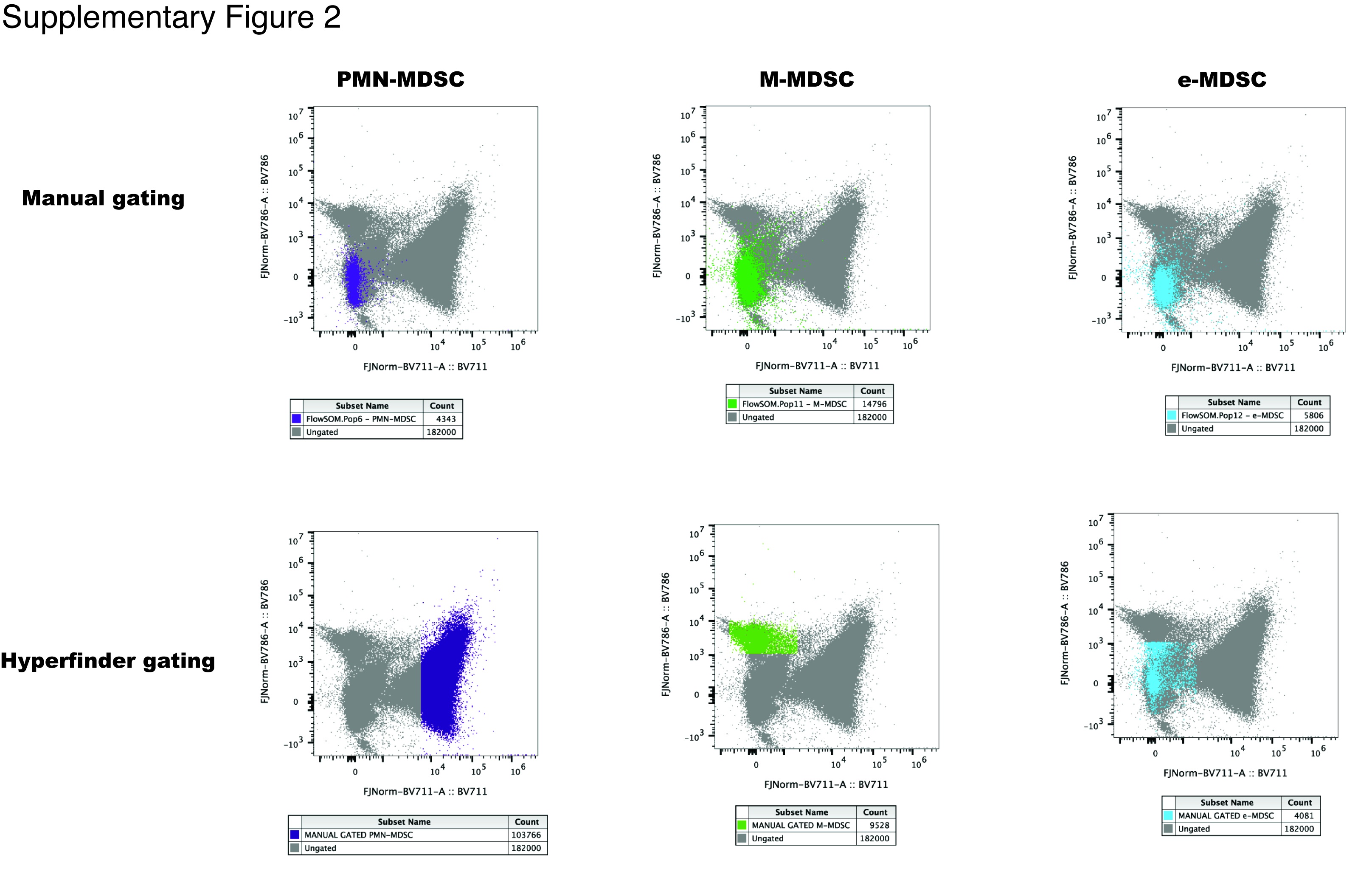

Supplement: Supplementary Figure 2 — Juxtaposition of MDSC clusters generated manually versus via supervised machine learning with FlowSOM algorithm followed by Hyperfinder gating. [file Image_2.tif]
