# Supplementary material for: Integrated machine learning approaches for flow cytometric quantification of myeloid-derived suppressor cells in acute sepsis
Source: Front Immunol. 2022 Nov 17;13:1007016. doi: 10.3389/fimmu.2022.1007016 (PMC9714638; doi:10.3389/fimmu.2022.1007016)
Supplement: Supplementary file 3 [file DataSheet_1.docx]

**Supplementary Material**

This supplement details installation of plugins into Flowjo v10.8.1, using an Apple Macintosh desktop computer running macOS Monterey v12.5, and R version 4.2.0 (4/22/2022). Equivalent software can be downloaded for users of Microsoft Windows operating system.

*R for macOS*

R is required for the FlowSOM plugin to function correctly. The R program can be accessed here: <https://cran.r-project.org/bin/macosx/>

The user should ensure that the Flowjo graphical user interface (GUI) searches for the R program in the correct folder. Open the Flowjo software, select **Preferences 🡪 Diagnostics 🡪 R path**. The R path should correspond to the location of the R program (e.g., /usr/local/bin/r).

*Plugins folder*

The user should ensure that the Flowjo GUI searches for the plugins listed below in the correct folder. Open the Flowjo software, select **Preferences 🡪 Diagnostics**. Ensure that the ‘Scan for plugins’ box is checked. Alternatively, manually enter the location of the plugins folder.

*Installation of Plugins*

All plugins were downloaded, free of charge from Flowjo Exchange ([www.flowjo.com/exchange/#/](http://www.flowjo.com/exchange/#/)) on June 10^th^, 2022.

The user should create a sub-folder in the ‘Applications’ folder, titled ‘plugins’. **Following installation of each plugin on the user’s operating system, the corresponding Java ARchive (.jar) file for each plugin should be moved into the ‘plugins’ folder created above.**

Correct installation of each plugin can be ensured in Flowjo by selecting **Workspace 🡪 Plugins**, which is a pull-down menu that lists all the correctly installed plugins and allows the user to execute the plugin via the GUI.

The following **plugin** **versions**, obtained from Flowjo Exchange, were used in this manuscript:

- DownSample version 3.3.1, published July 15^th^ 2021
  - No code available. This is BD proprietary.
  - Reference:  <https://docs.flowjo.com/seqgeq/dimensionality-reduction/downsample/>
- CyCombine version 1.0.0, published October 2022
  - GitHUB: https://github.com/biosurf/cyCombine
  - Reference: https://www.nature.com/articles/s41467-022-29383-5
- FlowSOM v3.0.18, published July 28^th^ 2021
  - GitHUB:  [https://github.com/SofieVG/FlowSOM](https://urldefense.com/v3/__https:/github.com/SofieVG/FlowSOM__;!!Ls64Rlj6!1OxwRPAjW3P1gU_TTZpHR0bIAFcj1hNULjH8fWvkVybXz-xMApF8HslEoaReJZgh8xgVtMHyw-Tk7r332x5j$)
  - Reference:  [https://onlinelibrary.wiley.com/doi/10.1002/cyto.a.22625](https://urldefense.com/v3/__https:/onlinelibrary.wiley.com/doi/10.1002/cyto.a.22625__;!!Ls64Rlj6!1OxwRPAjW3P1gU_TTZpHR0bIAFcj1hNULjH8fWvkVybXz-xMApF8HslEoaReJZgh8xgVtMHyw-Tk7ocNja1O$)
- UMAP v3.1, published March 25^th^, 2020
  - GitHUB:  [https://github.com/lmcinnes/umap](https://urldefense.com/v3/__https:/github.com/lmcinnes/umap__;!!Ls64Rlj6!1OxwRPAjW3P1gU_TTZpHR0bIAFcj1hNULjH8fWvkVybXz-xMApF8HslEoaReJZgh8xgVtMHyw-Tk7lcYIaq7$)
  - Reference:  [https://arxiv.org/abs/1802.03426](https://urldefense.com/v3/__https:/arxiv.org/abs/1802.03426__;!!Ls64Rlj6!1OxwRPAjW3P1gU_TTZpHR0bIAFcj1hNULjH8fWvkVybXz-xMApF8HslEoaReJZgh8xgVtMHyw-Tk7pCgDxIB$)
- ClusterExplorer v1.6.6, published April 18^th^ 2022
  - No code available. This is BD proprietary.
  - Reference: <https://docs.flowjo.com/flowjo/plugins-2/plugin-demonstration-videos/cluster-explorer/>
- Hyperfinder v0.6.8, published June 8^th^ 2021
  - No code available. This is BD proprietary.
  - Reference:  <https://www.bdbiosciences.com/content/dam/bdb/marketing-documents/BD-HyperFinder-White%20Paper.pdf>

*Generating the training model*

While the machine gating process requires minimal sample gating by the user, replication of the methods described in the manuscript require familiarity with the Flowjo GUI since the protocol described is only semi-automated.

1. Data extraction
   1. Each .fcs file (corresponding to a particular patient sample) was loaded into Flowjo, and fluorescent compensation was performed as per standard lab protocol. See Flowjo documentation for assistance in performing this step, if needed.
   2. Keywords were used to characterize/label each sample by using the Flowjo **Add Keyword** function. Keywords should be integers and should be sufficiently unique to identify each sample in the experiment. We assigned 2 unique keywords to each sample:
      1. Status (denoting the comparator groups: healthy, CINS or CIS were labeled 1,2 or 3 respectively due to the integer requirement)
      2. ID (denoting unique patient identifier)
   3. Each sample was then gated manually to exclude debris (FSC-A vs SSC-A), and to isolate singlets (FSC-A vs FSC-H).
   4. Each sample was gated for CD11b+, viable cells (CD11b-BV605 vs APC-Cy7) – see **Fig 1B** and **Supplementary Fig 1**
   5. **DownSample plugin** was manually executed on the CD11b+ population to extract 7000 events. This number was selected as it was the lowest common denominator amongst all stained samples, allowing equal number of events to be compared across samples.
   6. Gated events were exported as separate .fcs files as follows:
      1. **Right click** on the downsampled population in the Flowjo workspace 🡪 **Export/concatenate populations 🡪 Export 🡪 Parameters: All compensated parameters, Include Events: Include all 🡪 Export**
      2. Repeat the process to export equivalent, equally sized sub-populations of CD11b+, viable, singlets from each unique patient sample
   7. Open all exported .fcs files (from healthy, CINS and CIS patients) generated in step 1(e) in a new Flowjo workspace.
   8. Concatenate all exported files as follows:
      1. Select all .fcs files
      2. **Right click 🡪 Export/concatenate populations 🡪 Concatenate 🡪 Parameters: All uncompensated parameters, Group Concatenation: concatenate all files together, Additional Parameters: choose all keywords selected in step 1(b) 🡪 Concatenate**

***Note: failure to concatenate Keywords to the samples at this step will not allow samples to be uniquely identified following the concatenation step***

- - 1. This will generate a single file, composed of all concatenated files identified by their Keywords, in a separate workspace.

1. Batch correction
   1. cyCombine plugin was executed on the concatenated file. Batch, in our case, was identified by the ‘ID’ keyword, since each patient sample was analyzed on a separate day and therefore constituted a separate experiment, with associated technical variation (‘batch effect’).
2. Supervised clustering
   1. The batch-corrected .fcs file generated in Step 2 was then used for FlowSOM analysis **(Fig 1D)**. FlowSOM was executed as follows: **Workspace 🡪 Plugins 🡪 FlowSOM**. We manually fitted the model to the data by sequentially varying cluster number until we identified optimal fitting.

***Note: the version of FlowSOM installed in R, for these authors was FlowSOM v2.4.0 running igraph v1.3.2***

- 1. **UMAP (Fig 1E)** was executed as follows: **Workspace 🡪 Plugins 🡪 UMAP**. Select the cell markers that you wish to use for clustering.

***Note: UMAP settings used in our analysis were Euclidean, with 15 nearest neighbors and minimum distance 0.5.***

- 1. **ClusterExplorer (Fig 1F) (Workspace 🡪 Plugins 🡪 ClusterExplorer)** was executed to integrate UMAP and FlowSOM output. MDSC clusters were identified based on definitions of PMN-MDSC, M-MDSC and e-MDSC established *a priori.*

1. Optimized (machine) gating
   1. **Hyperfinder (Fig 1G)** was executed on each sub-population identified in step 2(c).

*Validating the machine workflow, and comparing to manual gating method*

One of the goals of the study was to compare manual gating to Hyperfinder-derived gates in the quantification of MDSC subtypes.

Hyperfinder gates can be applied to ‘Groups’ in a different Flowjo workspace. This will save the gating strategy and allow it to be applied to other samples. In order to apply the gating algorithm to other .fcs files, the Flowjo workspace containing these Groups is first saved as a **Workspace Template**. The gates will remain on the group in the Template and can be applied to other samples by opening the template and loading samples into that Group.

***Note: the cell markers used in .fcs files applied to Groups with Hyperfinder gating must have names that are identical to the cell markers used to generate the gates in Hyperfinder, for this strategy to work.***
